# Supplementary figures and images for: Adaptive Metropolis-coupled MCMC for BEAST 2
Source: PeerJ. 2020 Sep 16;8:e9473. doi: 10.7717/peerj.9473 (PMC7501786; doi:10.7717/peerj.9473)

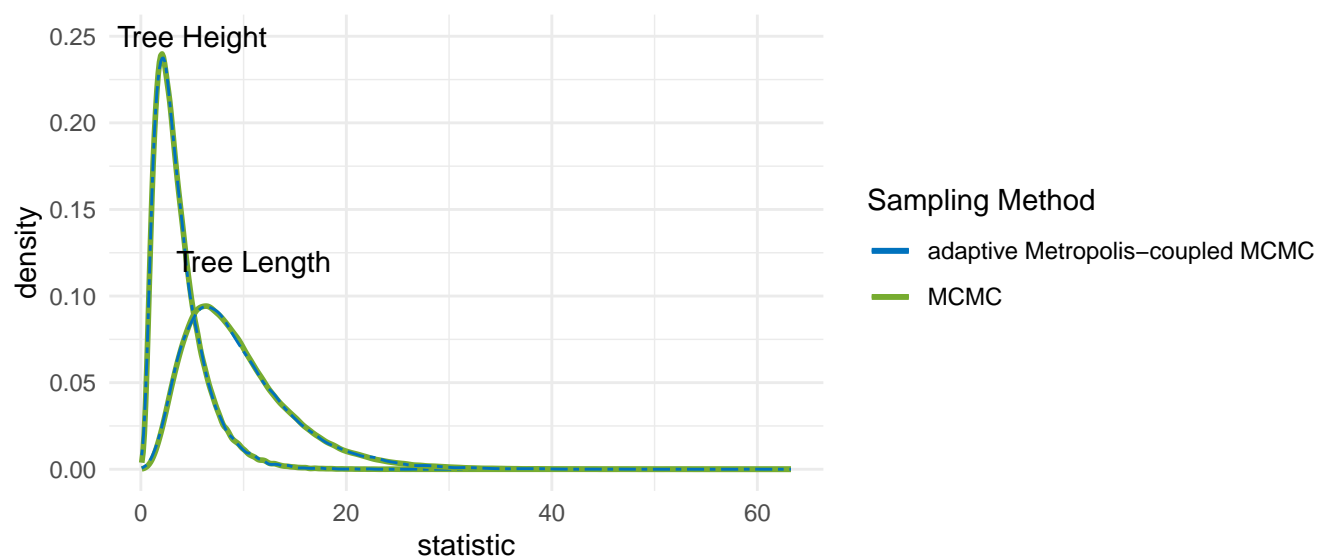

Supplement: Supplemental Information 1 — Comparison of the distribution of tree heights and tree lengths sampled under the structured coalescent using MultiTypeTree. The inferred distribution of tree heights and tree lengths match up between MCMC and the cold chain in MC3. [file peerj-08-9473-s001.pdf]

**A**

swap frequency = 100

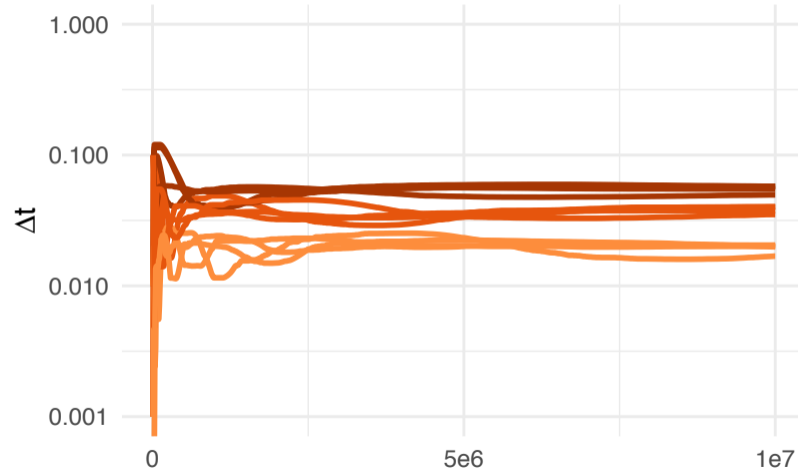**B**

swap frequency = 1000

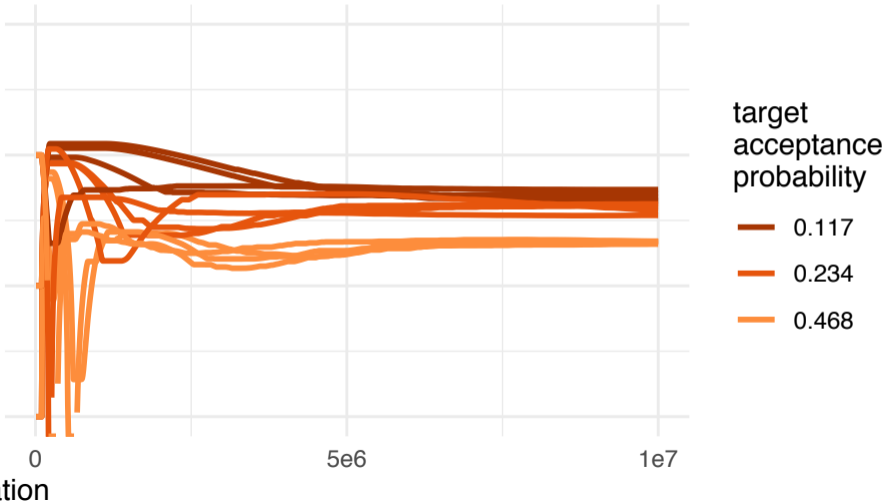

Supplement: Supplemental Information 3 — How the temperature difference between chains (y-axis) is adapted during the course of an adaptive parallel tempering run on the x-axis. Each colour represents runs with different target acceptance probabilities. For each of the four different target acceptance probabilities, we started runs at four different initial temperatures. A Acceptance probability over the course of a run when swaps of states between chains are proposed every 100 iteration. B Acceptance probability when swaps are proposed every 1000 iteration. [file peerj-08-9473-s003.pdf]

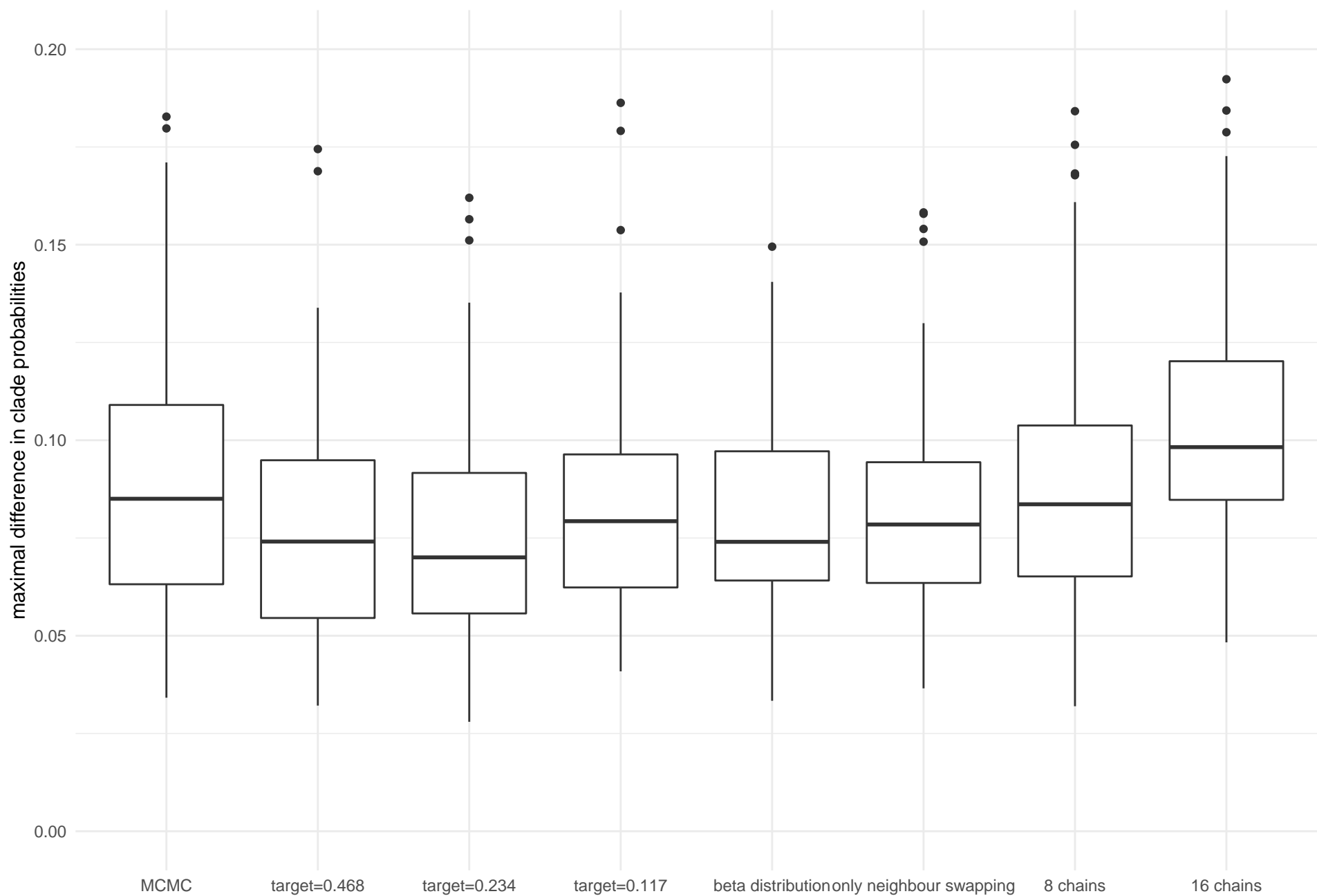

Supplement: Supplemental Information 5 — The distribution of maximal differences of clade supports for individual runs compared to a reference run. The reference run is made up of all 100 runs of an analysis. [file peerj-08-9473-s005.pdf]

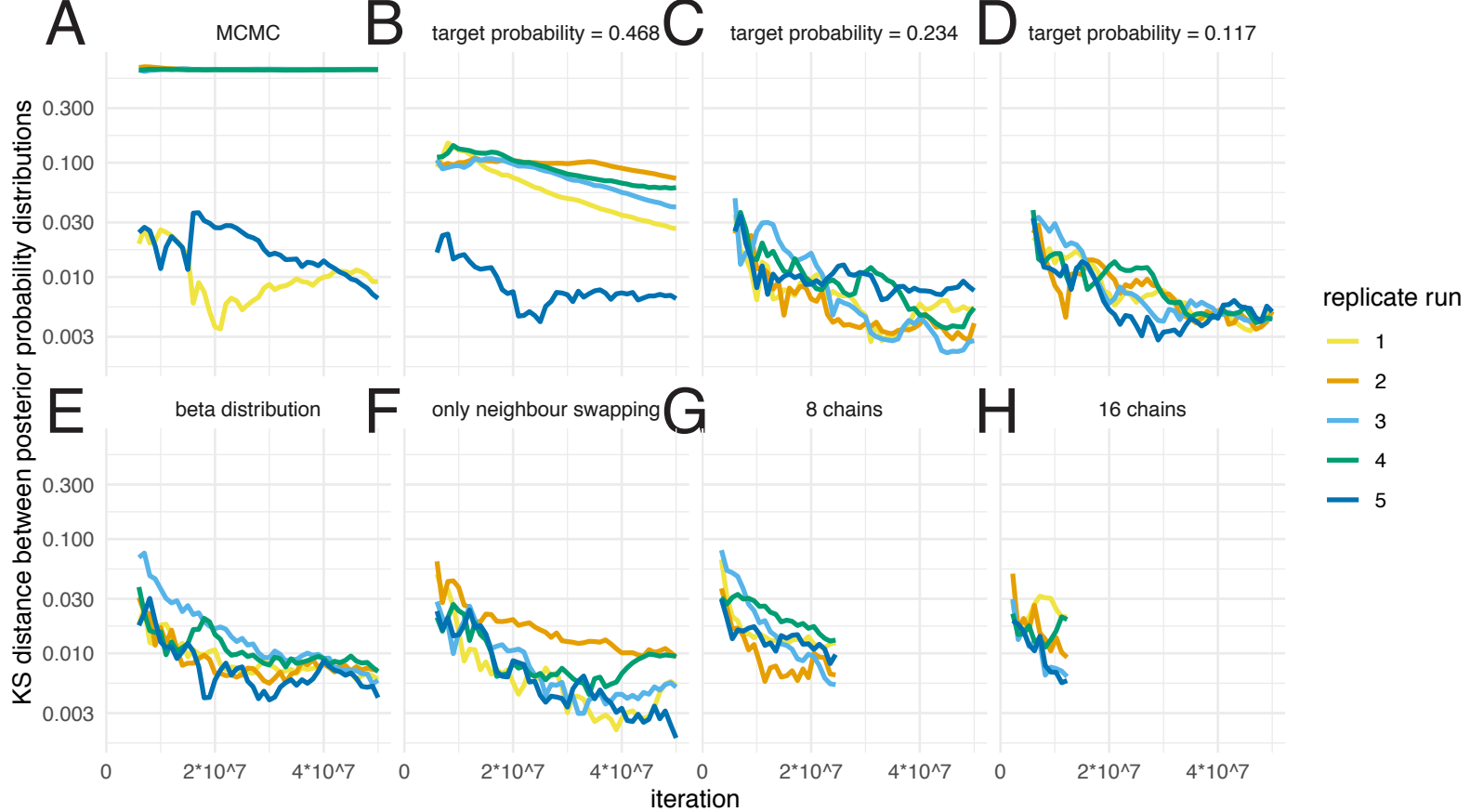

Supplement: Supplemental Information 6 — The Kolmogorov-Smirnov (KS) distance between the inferred posterior probability distribution and a reference posterior probability distribution on the y-axis up to the iteration on the x-axis. The different plots show the KS distance over the number of iterations for MCMC (A), and MC3 with a target acceptance probability of 0.468 B, 0.234 C and 0.117 D. In E, we assume the temperatures of the heated chains are distributed according to the quantiles of a beta distribution and a target acceptance probability of 0.234. In F, we only allow swaps between neighbouring chains and in G and H, we show the results when using 8 respectively 16 chains, but with only half respectively a quarter of the iterations. The reference distribution is made up of all MC3 runs that have a target acceptance probability of 0.234. In order to avoid comparing runs against themselves, we remove all runs with the same replicate number in the reference run for the KS calculation. [file peerj-08-9473-s006.pdf]
