# Supplementary material for: Adaptive Metropolis-coupled MCMC for BEAST 2
Source: PeerJ. 2020 Sep 16;8:e9473. doi: 10.7717/peerj.9473 (PMC7501786; doi:10.7717/peerj.9473)
Supplement: Supplemental Information 4 — The global acceptance probability during the course of an adaptive parallel tempering run on the x-axis. Each colour represents runs with different target acceptance probabilities. For each of the four different target acceptance probabilities, we started runs at four different initial temperatures. A Acceptance probability over the course of a run when swaps of states between chains are proposed every 100 iteration. B Acceptance probability when swaps are proposed every 1000 iteration. [file peerj-08-9473-s004.pdf]

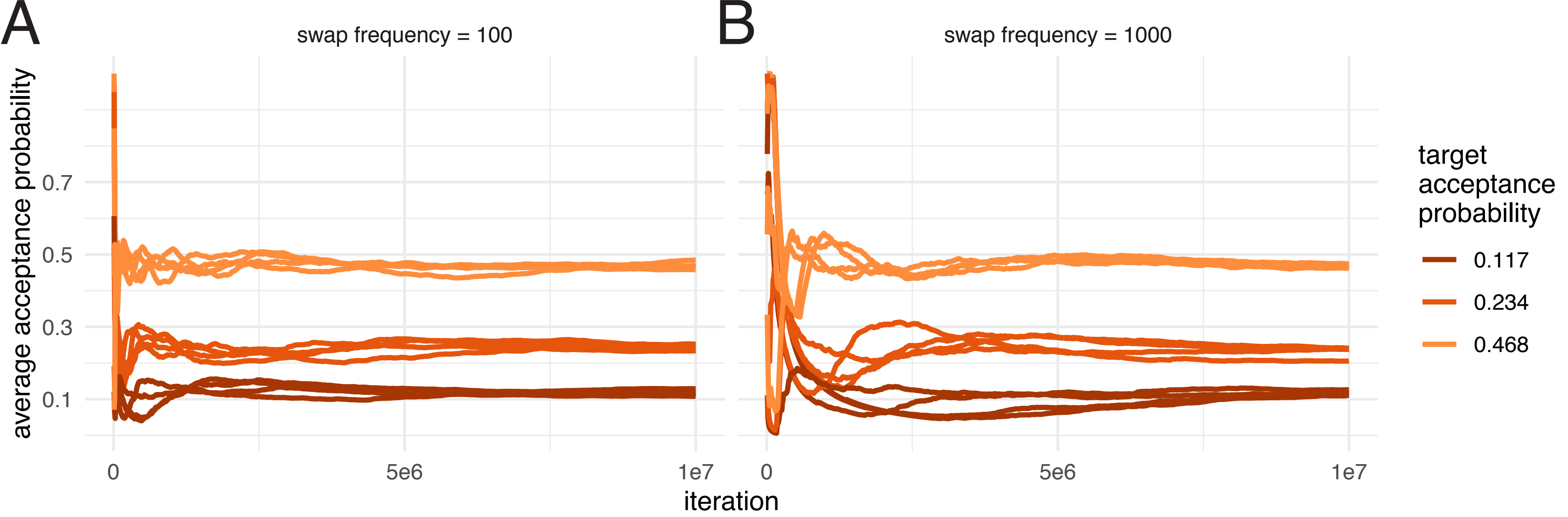
A
swap frequency = 100
B
swap frequency = 1000
average acceptance probability
0.7
0.5
0.3
0.1
0
5e6
1e7
iteration
target acceptance probability
0.117
0.234
0.468
